# Community-Based Surveillance and Geographic Information System‒Linked Contact Tracing in COVID-19 Case Identification, Ghana, March‒June 2020

**DOI:** 10.3201/eid2813.221068

**Published:** 2022-12

**Authors:** Ernest Kenu, Danielle T. Barradas, Delia A. Bandoh, Joseph A. Frimpong, Charles L. Noora, Franklin A. Bekoe

**Affiliations:** University of Ghana School of Public Health, Legon, Accra, Ghana (E. Kenu, D.A. Bandoh, J.A. Frimpong, C.L. Noora);; US Centers for Disease Control and Prevention, Accra (D.T. Barradas, J.A. Frimpong);; Ghana Health Service, Accra (F.A. Bekoe)

**Keywords:** COVID-19, community-based surveillance, geographic information system, GIS, case identification, contact tracing, districts, Ghana, coronavirus disease, respiratory infections, zoonoses

## Abstract

In response to the COVID-19 pandemic, Ghana implemented various mitigation strategies. We describe use of geographic information system (GIS)‒linked contact tracing and increased community-based surveillance (CBS) to help control spread of COVID-19 in Ghana. GIS-linked contact tracing was conducted during March 31–June 16, 2020, in 43 urban districts across 6 regions, and 1-time reverse transcription PCR testing of all persons within a 2-km radius of a confirmed case was performed. CBS was intensified in 6 rural districts during the same period. We extracted and analyzed data from Surveillance Outbreak Response Management and Analysis System and CBS registers. A total of 3,202 COVID-19 cases reported through GIS-linked contact tracing were associated with a 4-fold increase in the weekly number of reported SARS-CoV-2 infected cases. CBS identified 5.1% (8/157) of confirmed cases in 6 districts assessed. Adaptation of new methods, such as GIS-linked contact tracing and intensified CBS, improved COVID-19 case detection in Ghana.

The COVID-19 pandemic has elicited various responses to identify and control outbreaks and to save lives. Those responses include improving traditional outbreak investigation methods, enhancing surveillance, and developing vaccines (*1*–*3*). Ghana reported a case of COVID-19 in March 2020 and immediately activated response strategies. As of March 14, 2022, approximately 160,716 cases had been recorded (*4*), and the COVID-19 case-fatality rate was <1% (*5*), probably caused by interventions that were implemented to curb the spread of COVID-19 in this country (*6*).

Mitigation measures were implemented when the first 2 cases were recorded among persons with history of travel to an area experiencing a COVID-19 outbreak. These measures were a nationwide lockdown (*7*,*8*), contact tracing, widespread testing and reporting, and symptomatic treatment. Modern technology, such as use of smart phones to collect data on contacts and use of geographic information system (GIS) techniques in mapping out cases and contacts, were adopted to help improve existing surveillance methods. Subsequent detection of case-patients who did not have a travel history or apparent epidemiologic links to the initial cases, led to increased surveillance activities for early case detection and effective contact tracing at the community level (*7*).

As COVID-19 case-patients were isolated, symptomatically treated, and managed by case management teams, contacts of cases were identified and monitored for symptom development by using a 14-day COVID-19 symptoms diary and the Surveillance Outbreak Response Management and Analysis System (SORMAS) application, an electronic case-based outbreak investigation and response data collection and management tool (*9*). Symptomatic contacts were tested for SARS-CoV-2 and those who were positive were isolated and symptomatically treated.

A media campaign to heighten awareness and knowledge about COVID-19 was implemented across the nation by using radio and television. In periurban and rural areas, community-based surveillance (CBS) activities were also heightened (*8*). Community-based surveillance volunteers (CBSVs) were educated on COVID-19, its symptoms, and how to identify and report persons to the appropriate quarters (*10*).

Nevertheless, community transmission increased, and gaps in the SORMAS application and implementation architecture became more evident. Some of these gaps included difficulties in identifying the exact location of contacts and tracing them. As a result, the need for collection of case geolocation data became clear. In addition, unrestricted movement and travel in all other parts of the country also brought out the need for information on COVID-19 to be shared in hard-to-reach areas (*3*).

The routine surveillance focused on case-patients who sought ambulatory care at health facilities and their contacts listed. GIS-linked contact tracing, also known as enhanced contact tracing, is defined as a contact tracing based on spatial mapping of case-patients and contacts, active CBS, and household sampling and testing. GIS-linked contact tracing was implemented on March 31, 2020, in urban areas in Ghana. GIS was used to map documented COVID-19 cases; everyone who lived or worked within a certain distance was considered a possible contact. As an additional way to increase completeness of case identification, CBS was expanded in periurban, rural, and hard-to-reach areas (*6*).

Ghana is a country in West Africa located on the Atlantic Ocean. It shares borders with Burkina Faso to the north, Cote d’Ivoire to the west, and Togo to the east. The country has a population of ≈30 million persons, most (60.4%) of them having the working class ages of 15‒64 years (*11*). Because of its rich resources and development, the country has an average influx of 688,944 travelers each year (*12*). The country has a tiered health delivery system. The Ministry of Health serves as the policy directorate, and service delivery is provided through the Ghana Health Service, teaching hospitals, and other public and private agencies under the Ministry. We report the role GIS-linked contact tracing and CBS played in controlling the spread of COVID-19 in Ghana.

## Methods

### Study Setting and Population

The COVID-19 response in Ghana was implemented through a multisectoral approach with the president of the country leading the response by serving as chair of the Inter-Ministerial Coordinating Committee, a cross-government ministerial body that makes high-level decisions for swift response to the COVID-19 pandemic. The health sector response was led by the Ministry of Health with technical support from the National Technical Coordinating Committee and the National Public Health Emergency Operations Centre.

### GIS-Linked Contact Tracing

GIS-linked contact tracing is an advanced form of contact tracing in which mass testing is performed for of all contacts located within a specified distance from confirmed SARS-CoV-2‒infected cases. GIS-linked contact tracing was conducted during March 31–June 16, 2020, in 25 of the 29 urban districts starting in the Greater Accra region, which had the highest proportion of cases in the country at the time. GIS-linked contact tracing was extended to 18 of 29 districts in the Ashanti and Eastern Regions during April‒June 2020. In those areas, the Global Positioning System (GPS) coordinates of residences and places of employment of case-patients were collected and mapped. Collection and mapping were performed by using an ArcGIS web-based‒designed software (https://www.esri.com), which captured the coordinated the home or work location of the case-patient. A 2-km radius around each of the residences of the case-patient was used to define hotspots in which GIS-linked contact tracing would be conducted (*6*). To enable easy identification of persons within the targeted radius, movement within hotspots was also restricted by Security Services of Ghana including the police and military.

Surveillance officers visited households of confirmed case-patients and took GIS coordinates. After GIS was used to map confirmed COVID-19 cases upon identification, all persons who lived or worked within a 2-km radius of the home or work location of a case-patient, regardless of symptoms (*7*) or confirmed close exposure, were identified and considered possible contacts. Because this activity was conducted during the lockdown period, movement was restricted, making persons in the households easily accessible. Surveillance officers visited these households and collected nasopharyngeal specimens from all possible contacts within the demarcated radius and sent to the laboratory for testing by using reverse transcription PCR. Testing of possible contacts was completed within 48 hours after each case was confirmed and details shared with district health directorates.

Clinical specimens collected for SARS-CoV-2 testing were assigned unique barcodes, which were used to link contacts to their test results. GPS coordinates were also collected during specimen collection, and real-time data were generated as specimens were collected and tested. SARS-CoV-2 test results were uploaded into SORMAS by using the assigned barcodes. For persons who were positive for SARS-CoV-2, these newly identified case-patients were located by using coordinates and telephone details. Persons who were positive for SARS-CoV-2 were picked up by the country’s case management team (who came with ambulances and were fully donned in personal protective equipment) and sent to isolation centers for symptomatic treatment. Their close contacts were quarantined in their homes and monitored for 14 days by using the COVID-19 symptoms diary.

### Community-Based Surveillance

CBSVs are part of the Ghana Health Service disease surveillance structure and serve as a link between members of the community and the local health facility or district health directorate. They support community surveillance and provide up-to-date information on COVID-19 in the communities.

In rural, periurban, and hard-to-reach areas, COVID-19 cases were identified with the help of the existing CBS health structure. The district health directorate in 6 districts in the Ashanti, Western North, and Upper West Regions intensified activities of CBSVs during May–December 2020 by mobilizing volunteers and educating them about COVID-19, including details on the signs and symptoms of the disease. Those regions had highly active CBSVs who recently reported cases to their respective regions. The CBSVs from 6 districts in the 3 regions then embarked on household visits to conduct COVID-19 education and identify any suspected cases in their communities.

CBSVs identified any suspected COVID-19 case as defined as a person who had >1 of the following symptoms within the previous 14 days: fever, cough, shortness of breath, runny or stuffy nose, and headache. CBSVs documented and immediately reported the names of any suspected case-patient to their supervisor and referred the suspected case-patient to the nearest health facility. Name, place of work, place of residence, age, and telephone number of each suspected case-patient were recorded in the CBS register for follow-up, and nasopharyngeal swab specimens were collected from suspected case-patients by the district rapid response teams within 24 hours of identification of the suspected COVID-19 case.

Suspected case-patients were advised to quarantine until their results were made available to the district rapid response team (≈3‒4 days). Activities of CBSVs were monitored and analyzed for data completeness and response timeliness on a weekly basis by the district disease control officer to ensure all suspected cases they identified were duly reported to the district. Aggregate CBS register data were reported from the district health directorates to the national level monthly. To assess the contribution of CBS in COVID-19 case detection, we calculated the proportion of cases reported by CBSVs of the total number of cases detected at the district level.

### Data Extraction, Management, and Analysis

GIS-linked contact tracing data collected during March–June 2020 describing residential GPS coordinates, date of nasopharyngeal specimen collection, and SARS-CoV-2 test results for cases and contacts in the Greater Accra, Ashanti, and Eastern Region were extracted from SORMAS. The following data were extracted from monthly CBS reports submitted to the national level by the 6 districts during May 2020–December 2020: case-patient place of residence, GPS coordinates, modality of case identification, and test results. All data were cleaned and analyzed in by using Microsoft Excel 2016 (https://www.microsoft.com). Frequencies and proportions of cases detected through routine surveillance and CBS were calculated from both data sources. Heat maps were generated for GIS-linked contact tracing data by using ArcGIS.

### Ethics

This activity was part of the national pandemic preparedness response by the Ministry of Health, Ghana Health Service, and in accordance with Act 851 Public Health Act, 2012, Ministry of Health, Ghana. The Ghana Health Service Ethics Review Committee (GHS-ERC 006/05/20) also granted approval for use of data. Data were deidentified before extraction from the national databases to ensure that privacy of cases and contacts was not compromised. Data generated were stored electronically on national servers and password protected and were accessible only by the Ministry of Health, Ghana Health Service.

## Results

A total of 3,202 (average 200 cases/week) SARS-CoV-2‒infected case-patients were reported through GIS-linked contact tracing during March‒June 2020. Approximately 80%–90% of case-patient detected were asymptomatic. Before the GIS-linked contact tracing activity was implemented, the country had identified 193 (average 64 cases/week) positive cases during March 12‒31, 2020. The average weekly number of confirmed SARS-CoV-2‒infected case-patients increased 4-fold during GIS-linked contact tracing (Table 1).

**Table 1:** SARS-CoV-2‒infected cases identified through GIS-linked contact tracing, Greater Accra, and Eastern Regions, Ghana, March 31–June 16, 2020*

Modality	Before GIS-linked contact tracing, March 12–30, 2020	During GIS-linked contact tracing, March 31–June 16, 2020
No. contacts of known SARS-CoV-2‒infected persons who were reached for SARS-CoV-2 testing	653	86,248
No. SARS-CoV-2 tests conducted among contacts	651	85,463
No. SARS-CoV-2‒positive cases identified	193	3,202
Average weekly no. SARS-CoV-2‒infected cases identified	74	299

*Source: GHS 2020. COVID-19 sitrep March 2020. GHS, global health system; GIS, geographic information system.

**Figure 1:**
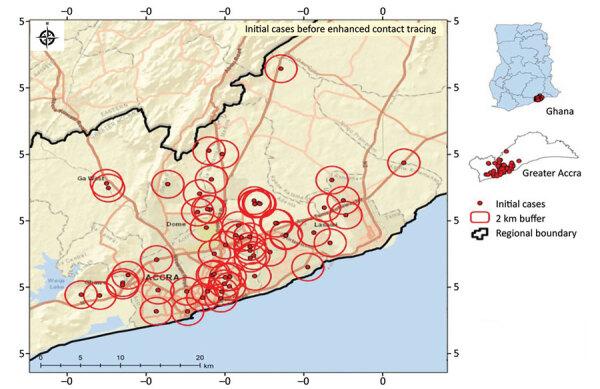
Sample spatial distribution of initial COVID-19 cases defining 2 km buffer around confirmed cases before geographic positioning system‒linked contact tracing, Greater Accra Region, Ghana, March 31, 2022. Insets show location of study area in Greater Accra and of Greater Accra in Ghana.

**Figure 2:**
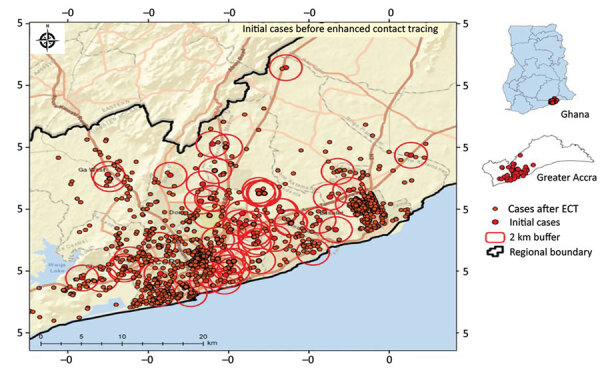
Sample spatial distribution of new COVID-19 cases identified after geographic positioning system‒linked contact tracing, Greater Accra Region, Ghana, May 16, 2020. Insets show location of study area in Greater Accra and of Greater Accra in Ghana. Large red circles indicate initial cases, and small red circles indicate cases after ECT. ECT, enhanced contact tracing.

Through GIS-linked contact tracing, we correctly identified the geolocation of residences of case-patients. With the known location of initial cases, new cases were identified near existing cases through mass testing to identify hotspot locations within the region (Figures 1, 2).

### Intensified CBS

In the 6 districts in which CBS activities were intensified and assessed, 157 SARS-CoV-2 cases were reported through routine surveillance or CBSVs. These volunteers reported 5.1% (8/157) of all confirmed SARS-CoV-2 cases that were all in hard-to-reach communities (Table 2). In these 6 selected districts, all 157 case-patients detected were followed-up by the district and regional rapid response teams for identification of contacts, contact tracing, and referral to medical care. Most (60%) contacts of case-patients in the district after detection were also followed-up by CBSVs for symptoms monitoring.

**Table 2:** Comparison of SARS-CoV-2‒infected persons (cases) identified through routine surveillance and CBS in 6 districts, Ghana, May 1–December 31, 2020*

District	No. cases reported from district	No. cases detected by CBS	Proportion reported by CBS volunteers, %
Amansie Central	60	3	5.0
Bia East	8	1	12.5
Bosome Freho	63	4	6.3
Sefwi Akontonbra	3	0	0.0
Sissala East	7	0	0.0
Sissala West	16	0	0.0
Total of all districts	157	8	5.1

*CBS, community-based surveillance.

## Discussion

We report the role that GIS-linked contact tracing and CBS played in detection of COVID-19 cases in Ghana, including asymptomatic cases during the early phase of the pandemic. Those procedures probably assisted in containing the spread of COVID-19 in Ghana. The number of persons who had suspected COVID-19 and were identified for SARS-CoV-2 testing after introduction of GIS-linked contact tracing increased from 63 (average 21/week) to 86,248 (average 5,390/week) persons. The number of positive cases increased from an average of 64 cases/week to an average of 200 cases/week, and the geographic distribution of the cases was more widespread than before GIS-linked contact tracing was adopted. The GIS-linked contact tracing provided the opportunity to identify other persons who were exposed through community transmission and had SARS-CoV-2 infection develop.

Involvement of CBSVs in tracking contacts of cases during the COVID-19 response might have led to identification of SARS-CoV-2‒infected case-patients, which probably would have been missed by traditional facility-based passive surveillance (*13*) because many of these case-patients were asymptomatic (*14*). A large number of case-patients were reached, tested, and recorded outside healthcare facilities as part of CBSVs-assisted and GIS-linked contact tracing and case identification efforts. Timely identification and isolation of cases probably helped reduce further community transmission that would have occurred in the country (*3*).

Despite the positive effect of GIS-linked contact tracing and use of CBSVs, cost implications threaten its sustainability. In Ghana, these implications included shortages of consumables for testing, inadequate human resources to meet the high workload, and other factors such as vehicular challenges. To mitigate some of these setbacks, limited resources were channeled to identify communities with high burden of COVID-19 in which action was needed to contain the spread of SARS-CoV-2 infection.

The contribution of CBS activities in the hard-to-reach areas and districts far away from hotspot areas demonstrates how their activities helped in preventing community transmission and containment in those districts. CBSVs supported the health system in conducting contact tracing follow-up visits at the community level. Using CBS is a cost-effective strategy for managing community health-related activities because CBS is not given any renumeration. CBS uses persons selected by their communities to offer voluntary services in hard-to-reach areas. Given that 5% of the cases in hard-to-reach areas were identified by CBSVs, including these persons in the health structure is advantageous. To maximize the benefit of CBS, providing targeted training and ensuring that they work closely with health workers in these areas are essential.

Some limitations of this report include the inability to attribute increases in case finding solely to GIS-linked contact tracing or CBSVs because there were also attempts to increase awareness and testing through media. Because spread of COVID-19 also increased over time (despite mitigation and containment efforts), there were generally more persons to find and test. Data loss during the GIS-linked contact tracing implementation period precluded analysis of case-level data. Thus, we are unable to report on indicators such as age, sex, district, and district-specific testing yields. Because GIS-linked contact tracing was implemented in all districts in the selected regions, comparative data during the same period are not available. Despite those limitations, GIS-linked contact tracing and CBS apparently contributed to case finding during the early phases of the COVID-19 epidemic in Ghana. Those 2 response strategies were believed to be crucial to early containment efforts and might have contributed to the slow spread of COVID-19 in participating districts during the first 3 months of the epidemic in Ghana.

Application of enhanced surveillance in Ghana has identified the need to prioritize geospatial data in surveillance activities. Using real-time surveillance to provided specific information during a public health emergency has led to identifying opportunities to build the capacity of surveillance staff in geospatial mapping. Through this approach, a new and improved path for surveillance and response in Ghana has been created. Geospatial data can improve targeted responses in emergency situations leading to better use of limited resources that might be available. GIS-linked contact tracing and community-based surveillance, as part of the overall strategy for combating COVID-19 in Ghana, were beneficial in identification of SARS-CoV-2‒infected cases within affected communities, particularly asymptomatic cases that might have been missed by passive health facility‒based surveillance approaches.
